# SIRT3 Acetylation Regulates Mitophagy to Alleviate Deoxynivalenol-Induced Apoptosis in Porcine Alveolar Macrophages Cells

**DOI:** 10.3390/ijms26178222

**Published:** 2025-08-25

**Authors:** Peng Fan, Huidan Deng, Ya Wang, Zhihua Ren, Junliang Deng

**Affiliations:** 1 Key Laboratory of Animal Disease and Human Health of Sichuan Province, College of Veterinary Medicine, Sichuan Agricultural University, Chengdu 611130, China; 2022203062@stu.sicau.edu.cn (P.F.); denghuidan@sicau.edu.cn (H.D.); wangyayang@126.com (Y.W.); 2 College of Veterinary Medicine, Henan Agricultural University, Zhengzhou 450046, China

**Keywords:** SIRT3, DON, acetylation, mitophagy, apoptosis, PAM

## Abstract

Deoxynivalenol (DON), a global mycotoxin contaminant, induces immunotoxicity in swine and humans by disrupting mitochondrial membrane integrity and activating mitophagy. SIRT3 plays an important role in regulating cell metabolism and various diseases. It also regulates apoptosis (caused by DON) by regulating the mitophagy pathway, but this pathway has not been studied yet. Gene knockout and overexpression of SIRT3 were performed for proteomics and acetylation modification. Therefore, in this study, PAM cells were selected as an in vitro model of DON (1.1 μg/mL) exposure for 24 h. The results showed that the knockout impaired mitochondrial antioxidant function, whereas overexpression improves damage stimulation. DON can also affect the metabolism of immune pathways, but SIRT3 can enrich these substances’ metabolism. The results of the acetylation modification analysis showed that knockout affected the mRNA metabolism and others, while overexpression affected apoptosis and others. DON exposure caused fatty acid degradation, and altered MAPK signaling pathway. Knockout and overexpression of SIRT3 under DON exposure were enriched in PPAR, Ferroptosis pathway. Overexpression attenuated DON-induced mitophagy by reducing cellular ROS, as well as the expression of LC3, P62 and PINK1/Parkin. Finally, SIRT3 reduced cell apoptosis by reducing the expression of BAX and CASP3 and increasing the expression of BCL-2. These results indicated that SIRT3 could alleviate DON-induced cell damage by reducing apoptosis through the mitophagy pathway.

## 1. Introduction

Deoxynivalenol (DON) is a type B trichothecene that has been the most prevalent mycotoxin in raw feed materials and byproducts in recent years [1]. However, this can cause serious problems in the final consumers of feed, such as gastrointestinal toxicity, immunotoxicity, hepatorenal toxicity, neurotoxicity, carcinogenicity, teratogenicity and mutagenicity. Immunotoxicity is one of the most harmful diseases in pigs [2]. Furthermore, DON causes immunotoxicity by binding to the 60S subunit of the eukaryotic ribosome and blocking mRNA translation initiation [3], leading to ribosomal stress and apoptosis [4]. DON can also induce the accumulation of mitochondrial reactive oxygen species (ROS), increase oxidative stress, destroy the mitochondrial membrane structure, and increase mitophagy [5]. Subsequently, the expression of the regulatory genes Bax, Bak-1 and Caspase-3 is altered, which ultimately activates apoptosis-related pathways [6], leading to cell damage.

Mitochondria are the most important organelles in eukaryotic cells because they not only provide energy for cell activities through oxidative phosphorylation but also participate in the regulation of cell metabolism, stress signal transduction [7], calcium homeostasis, reactive oxygen species production and apoptosis [8]. When damaged or aged, mitochondria can be degraded and recycled via mitophagy [9]. At present, there are two main pathways of mitophagy: ubiquitin-mediated mitophagy and autophagy receptor-mediated mitophagy [10,11]. Among them, the ubiquitin pathway (selective autophagic degradation of damaged mitochondria), which involves mainly PINK1/Parkin, is one of the most intensively studied pathways. PINK1 can accumulate on the mitochondrial surface and induce the translocation of Parkin, P62 and LC3 from the cytoplasmic matrix to the mitochondria, which ultimately leads to mitophagy [12]. When mitochondria are affected by toxins, stress, etc., mitophagy activity is enhanced, which accelerates apoptosis by activating the proapoptotic protein BAX, of the BCL-2 family, the main effector of apoptosis, the formation of apoptotic bodies and the activation of caspase-3 [13].

Silent mating type information regulation 2 homolog 3 (SIRT3) is a NAD^+^-dependent lysine deacetylase located in mitochondria that regulates a variety of cellular processes. These include metabolism [14], neuroprotection [15], cell proliferation [16] and apoptosis regulation [17]. So far, SIRT3 has been widely studied because it is related to many systemic diseases, such as those of the nervous system and cardiovascular system, kidney disease and tumors. SIRT3 can directly upregulate the key regulatory proteins of mitophagy, Bnip3 (Bcl-2/E1B 19 kDa protein-interacting protein 3) and Nix (Bcl-2/E1B 19 kDa interacting protein 3-like), and MAP1LC3 (microtubule-associated protein 1 light chain 3, LC3) levels regulate mitophagy [18]. In addition, SIRT3 can inhibit cell apoptosis by deacetylating P53, weakening the binding of P53 to the Bcl-2-associated immortalized protein 2 (BAG2) complex, and activating the bcl-2-related antiapoptotic pathway [19]. However, current research on SIRT3 and mitophagy mainly focuses on human and mouse models, and there application of SIRT3 in the pig system has been reported yet.

Protein posttranslational modifications (PTMs) play crucial roles in various physiological and pathological processes, contributing to almost all biological pathways. Protein deacetylation is one of the major mitochondrial posttranslational modifications [20] and regulates various metabolic processes in mitochondria by affecting the activity and function of mitochondrial enzymes [21]. SIRT3 can indirectly affect PINK1/Parkin pathway activity by maintaining the mitochondrial membrane potential [22], thereby attenuating mitophagy caused by a decrease in the membrane potential [23]. SIRT3 also inhibits the expression of the proapoptotic protein Bax through deacetylation while promoting the activity of the antiapoptotic protein Bcl-2 [24], preventing the activation of the caspase cascade, significantly reducing the activity of CASP3, regulating all aspects of apoptosis [25] and alleviating apoptosis.

Porcine Alveolar Macrophages (PAMs) are the core immune cells that colonize the alveolar space of porcine lungs and play a key role in the immune system [26]. However, the regulation of SIRT3 expression in DON-exposed PAM cells is still unclear, and the effect of SIRT3 on DON-induced acetylation in PAM cells has not yet been reported. Therefore, in this study, proteomics and acetylomics analyses were used to detect the differential protein expression and differentially acetylated peptide expression of SIRT3 in PAM cells exposed to DON. SIRT3 was shown to reduce DON-induced apoptosis, potentially through mitophagy pathway regulation. This study provides a theoretical basis for further research on the targeted regulation of SIRT3 in mycotoxins.

## 2. Results

### 2.1. Model Construction and the Effect of DON on PAM Cell Viability

**Figure 1:**
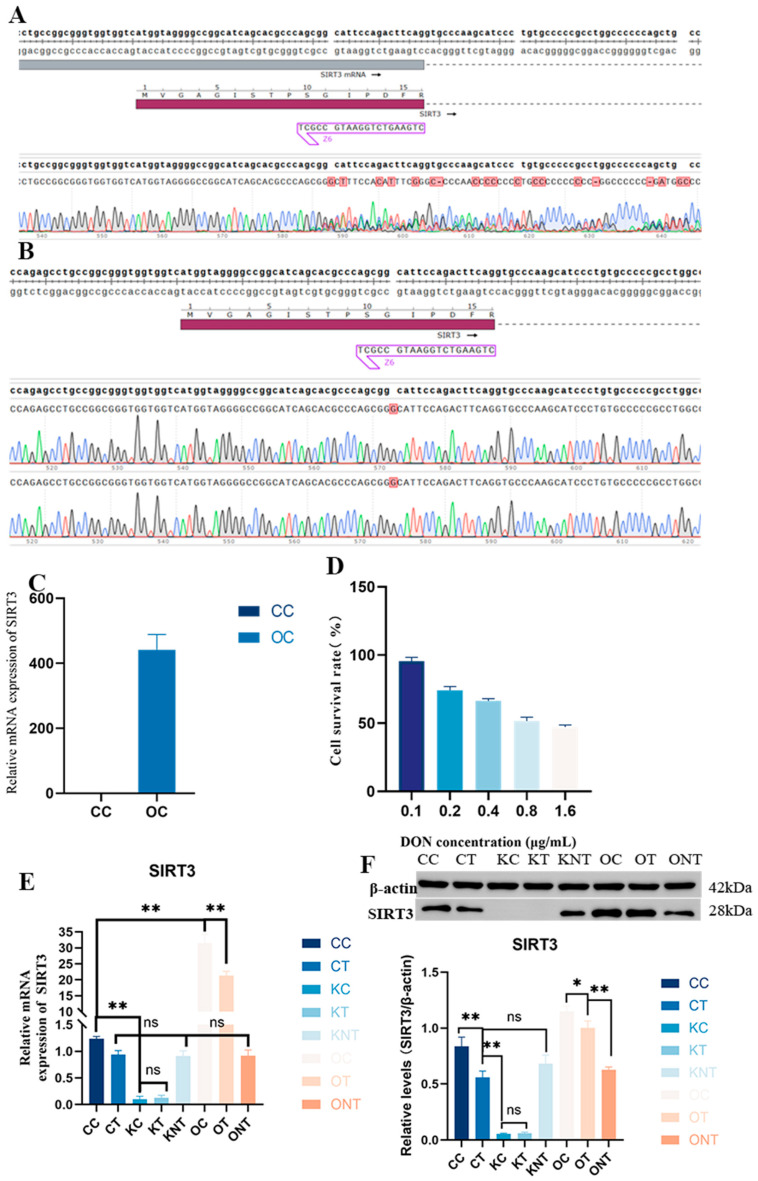
**Model construction and the effect of DON on PAM cell viability.** (**A**,**B**). The first and second sequencing results for knockout cells showed that base *G* was inserted and nesting peaks appeared, (**C**). Relative mRNA expression of SIRT3, (**D**). Effect of DON on the PAM cell survival rate, (**E**). The relative mRNA expression levels of SIRT3, (**F**). Protein expression levels of SIRT3, and a statistical analysis graph depicting these protein expression levels was generated using Image J (1.53r) software expression at the protein levels. Data are presented as mean ± SE (n = 3). Significance compared with control (ns *p* > 0.05, * *p* < 0.05, and ** *p* < 0.01). The same applies in subsequent figures. The arrows indicate the direction of replication, and different colors represent different bases.

The results of two sequencing verifications of the knockout model are shown in Figure 1A,B and Supplemental Figure S1A. The results revealed that the target site was inserted into base G, and there was a nesting peak, a frameshift mutation and a stop codon at the 39th amino acid, indicating that the monoclonal clone was successful. The results of the verification of the overexpression enzyme digestion and plasmid sequencing are shown in Figure 1C and Supplemental Figure S1B, indicating that the strong promoter was transferred. Subsequently, PAM cells infected with lentivirus were verified via qPCR, and the results revealed that SIRT3 expression was increased. The effect of DON on the PAM cell survival rate was subsequently evaluated, as shown in Figure 1E. The IC_50_ of the DON for PAM was calculated as 1.1 μg/mL. Finally, we detected the expression of the SIRT3 gene and protein in cells exposed to DON via WB and RT-qPCR. The detailed grouping is provided in Supplementary Table S1. As shown in Figure 1F, DON exposure caused a decrease in SIRT3, but not significantly. After the overexpression of SIRT3, the expression of SIRT3 significantly increased. This indicated that the model was successfully constructed.

### 2.2. Proteomics Quality Control

We performed a proteomic analysis to investigate further the effects of SIRT3 deletion and overexpression on total PAM protein expression under DON exposure. As shown in Supplemental Figure S2A–D, Pearson’s correlation coefficient (PCC), principal component analysis (PCA), relative standard deviation (RSD) and protein abundance analysis indicated strong correlation and reproducibility of the duplicate samples. A total of 10,172 proteins were identified via proteomic analysis. The statistical results for the differential proteins between groups are shown in Supplemental Figures S2E,F and S3, which show the significant differences in proteins between the comparison groups.

### 2.3. Bioinformatics Analysis of the Proteomic Data

**Figure 2:**
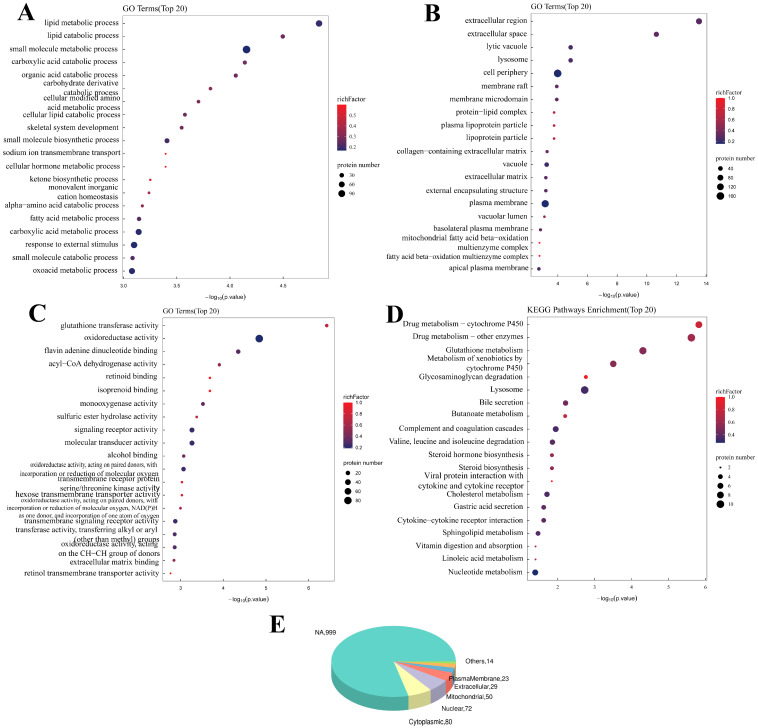
**Bioinformatics analysis of intracellular differential proteins in PAM under the influence of knockout SIRT3 (KC vs. CC)**. (**A**): GO-BP enrichment bubble plot (KC vs. CC), (**B**): GO-CC enrichment bubble plot (KC vs. CC), (**C**): GO-MF enrichment bubble plot (KC vs. CC), (**D**): KEGG pathway classification map (KC vs. CC), (**E**): Pie chart of subcellular localization distribution. Enriched GO terms with a *p*-value of <0.05 are exhibited (n = 3).

As shown in Figure 2, the GO enrichment analysis after SIRT3 was knocked out revealed that SIRT3 was related to the Biological Process (BP) of the metabolism of various substances, such as lipids and carbohydrates. In addition to the Molecular Function (MF) of glutathione transferase activity, oxidoreductase activity, flavin adenine dinucleotide binding and others, the Cellular Component terms of lytic vacuoles, lysosomes, the cell periphery and others were also significantly correlated. The KEGG analysis revealed that these genes were enriched in drug metabolism, glutathione metabolism, glycosaminoglycan degradation and other pathways.

The GO enrichment analysis after SIRT3 overexpression revealed that the differentially expressed proteins were related to the regulation of body fluid levels, the regulation of the response to sodium, the response to external stimuli and other BPs (Supplemental Figure S4). In addition, Cellular Components such as protein–lipid complexes, lipoprotein particles and plasma lipoprotein particles and molecular functions such as isoprenoid binding, retinoid binding and the transmembrane transfer activity of various substances were significantly related. The KEGG analysis revealed that these genes were enriched mainly in adrenergic signaling in cardiomyocytes, arachidonic acid metabolism, mineral absorption and other pathways.

**Figure 3:**
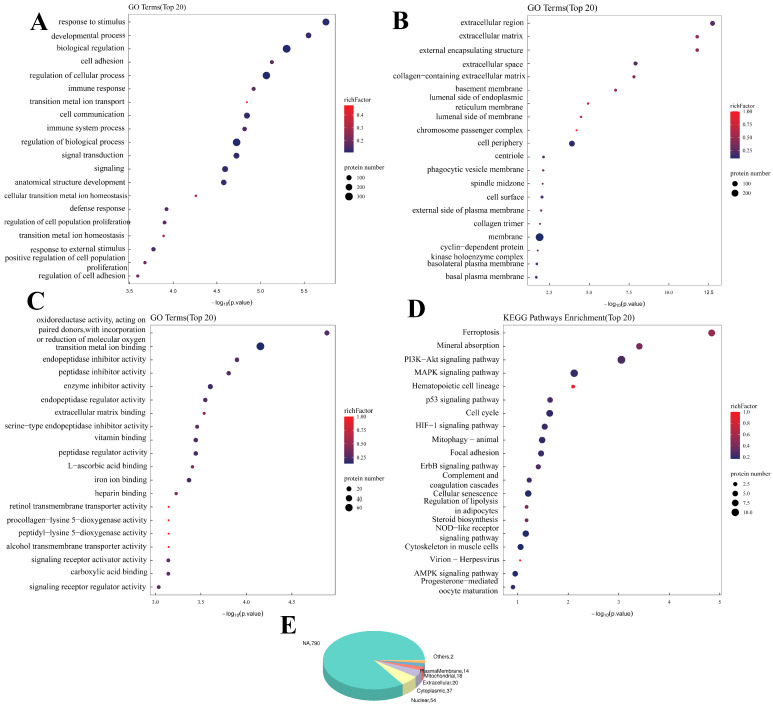
**Bioinformatics analysis of intracellular differential proteins in PAM under the influence of DON (CT vs. CC).** (**A**): GO-BP enrichment bubble plot (CT vs. CC), (**B**): GO-CC enrichment bubble plot (CT vs. CC), (**C**): GO-MF enrichment bubble plot (CT vs. CC), (**D**): KEGG pathway classification map (CT vs. CC), (**E**): Pie chart of subcellular localization distribution. Enriched GO terms with a *p*-value of <0.05 are exhibited (n = 3).

The DEPs affected by DON were related mainly to BPs (Figure 3), such as response to stimulus, developmental process and biological regulation. They were also related to Cellular Components, such as the extracellular region, external encapsulating structure and extracellular matrix. These proteins are also associated with oxidoreductase activity, act on paired donors and incorporate or reduce molecular oxygen, endopeptidase inhibitor activity and other MFs, and the KEGG analysis revealed that the differentially expressed proteins were enriched mainly in ferroptosis, the cell cycle, mitophagy and other pathways.

The DEPs between DON and PAM after SIRT3 knockout, such as lytic vacuoles, lysosomes and cell peripheries in terms of Cellular Components, were also related to the metabolic process, whereas such as various peptidase activities in MFs were altered. The KEGG enrichment analysis also revealed the metabolic pathways associated with each substance.

However, the DEPs affected by DON after SIRT3 overexpression were related mainly to BPs, such as the regulation of the response to wounding, wound healing, complement activation and the classical pathway. Cellular Components such as haptoglobin–hemoglobin complexes, external encapsulating structures, membrane endopeptidase activity and other MFs were also significantly correlated. The KEGG analysis revealed that these genes are involved mainly in the complement, coagulation cascades. Other pathways were also enriched.

Finally, subcellular localization annotation revealed that the proteins affected by either SIRT3 or DON were located mainly in the nucleus, cytoplasm, extracellular space and mitochondria. Taken together, both DON addition and SIRT3 gene editing significantly affected immune stimulation and metabolism-related pathways.

### 2.4. Quality Control of Acetylation Omics

We also examined the acetylation level under the influence of SIRT3 and DON in the subsequent experiments, and the results are statistically analyzed in Supplemental Figures S7 and S8. The mass deviations of all the identified acetylated peptides were distributed within 10 ppm, and the Andromeda score was ideal, indicating that the identification results were accurate and reliable and that high-quality experimental data were obtained. A total of 3196 acetylated proteins, 3624 acetylated peptides and 6468 acetylation sites were identified in this study, of which 3192 modified proteins had 3609 quantitatively acetylated peptides and 6450 quantitatively acetylated sites. The number of acetylation sites on all the identified proteins was then counted. The statistical results revealed that 35.7% of the proteins had two or more modification sites, and the average number of acetylation sites per 100 amino acids was 16.75. Significant differences in acetylated peptides between groups are shown below (cluster plot or volcano plot). In addition, the conserved amino acid motifs of the acetylated modification sites on the modified peptides were analyzed. The results showed that lysine (K) was most easily acetylated under the DON or SIRT3 treatment.

### 2.5. Bioinformatics Analysis of the Acetylation Omics

**Figure 4:**
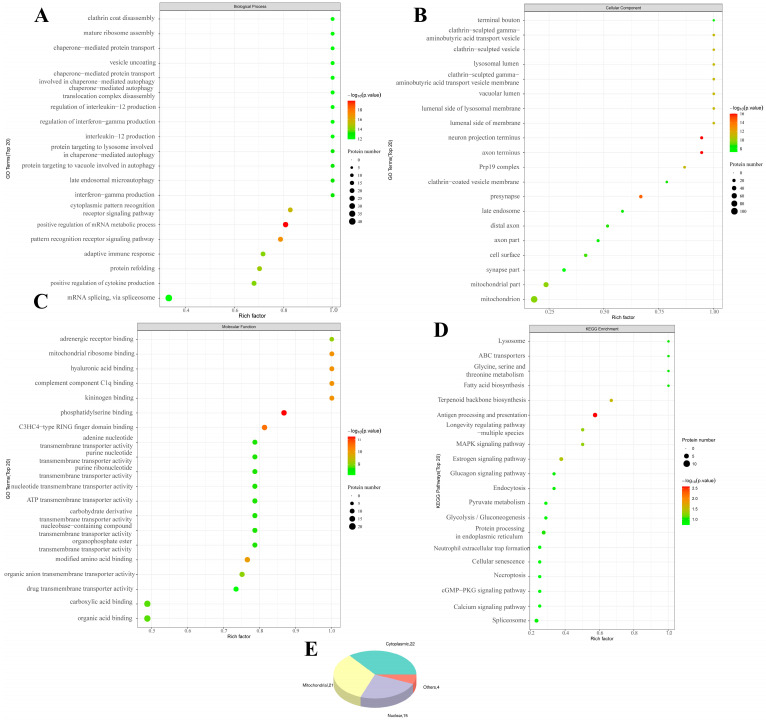
**Bioinformatics analysis of intracellular differentially modified acetylated peptides of PAM under the influence of knockout SIRT3 (KC vs. CC)**. (**A**): GO-BP enrichment bubble plot (KC vs. CC), (**B**): GO-CC enrichment bubble plot (KC vs. CC), (**C**): GO-MF enrichment bubble plot (KC vs. CC), (**D**): KEGG pathway classification map (KC vs. CC), (**E**): Pie chart of subcellular localization distribution. Enriched GO terms with a *p*-value of <0.05 are exhibited (n = 3).

We then performed a bioinformatics analysis of the differentially modified peptides (Figure 4 and Supplemental Figure S9). GO enrichment analysis revealed that SIRT3 knockout was associated with positive regulation of biological processes (mRNA metabolic process, PRRs, cytoplasmic pattern recognition receptor signaling pathway and others), cellular components (neuron projection terminus, axon terminus and presynapse), and molecular functions (phosphatidylserine binding, C3HC4-type RING finger domain binding, hyaluronic acid binding and others). KEGG enrichment analysis revealed that these peptides were enriched in antigen processing and presentation, terpenoid backbone biosynthesis, estrogen signaling pathway and other pathways.

We then performed a bioinformatics analysis of the differentially modified peptides. The GO enrichment analysis after SIRT3 overexpression revealed negative regulation of mitochondrial organization, negative regulation of the extrinsic apoptotic signaling pathway and regulation of protein localization to the nucleus and other BPs. On the other hand, Cellular Components are intermediate filaments, intermediate filament cytoskeletons and nuclear lamina. Finally, the MFs included phosphatidylserine binding, modified amino acid binding, C3HC4-type RING finger domain binding, etc. KEGG enrichment analysis revealed significant changes in the cytoskeletons of muscle cells and thermogenesis and protein processing in endoplasmic reticulum pathways.

**Figure 5:**
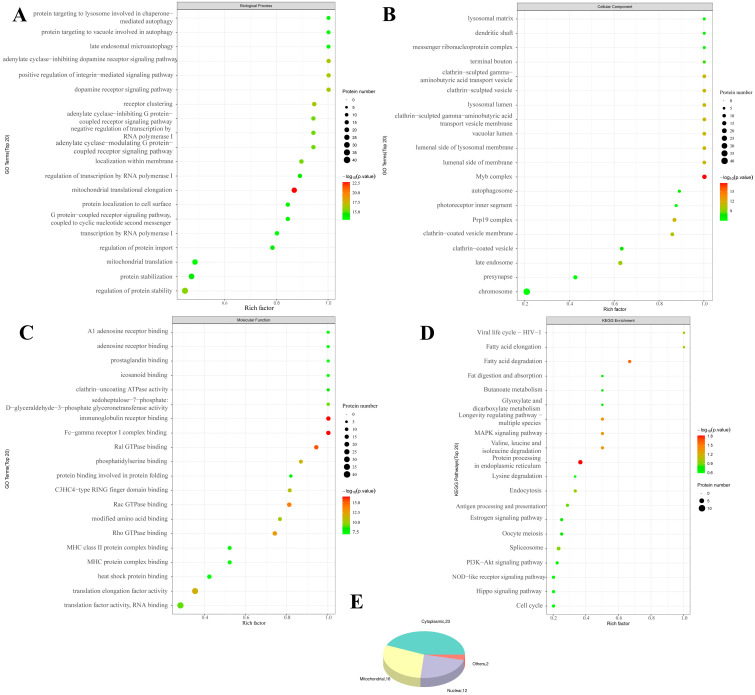
**Bioinformatics analysis of intracellular differentially modified acetylated peptides of PAM under the influence of DON (CT vs. CC)**. (**A**): GO-BP enrichment bubble plot (CT vs. CC), (**B**): GO-CC enrichment bubble plot (CT vs. CC), (**C**): GO-MF enrichment bubble plot (CT vs. CC), (**D**): KEGG pathway classification map (CT vs. CC), (**E**): Pie chart of subcellular localization distribution. Enriched GO terms with a *p*-value of <0.05 are exhibited. (n = 3).

The differentially modified peptides (Figure 5) affected by DON were related mainly to mitochondrial translational elongation and positive regulation of the integrin-mediated signaling pathway, adenylate cyclase-inhibiting dopamine receptor signaling pathway and other BPs, whereas the Myb complex, Prp19 complex and lumenal side of the lysosomal membrane and other Cellular Components were affected. Mitochondrial translational elongation, Fc-gamma receptor I complex binding, immunoglobulin receptor binding and other MFs also changed significantly. KEGG analysis revealed that the differentially modified peptides were enriched in protein processing in the endoplasmic reticulum, the MAPK signaling pathway, the longevity regulating pathway and other pathways.

The differentially modified peptides of DON on the PAM after SIRT3 knockout were also related mainly to mitochondrial translational elongation and positive regulation of the integrin-mediated signaling pathway and BPs, such as the metabolic process of drugs, tricarboxylic acid, antibiotics, cofactors and many other substances. The activity of the Myb complex, mitochondrion, mitochondrial matrix and other proteins with mitochondrial localization, succinate-CoA ligase (GDP-forming) activity, Fc-gamma receptor I complex binding, immunoglobulin receptor binding and other MFs also significantly changed. KEGG analysis revealed that the differentially modified peptides were enriched mainly in arginine and proline metabolism, the PPAR signaling pathway and other pathways.

However, the differentially modified peptides affected by DON after the overexpression of SIRT3 were enriched mainly in primary miRNA processing, miRNA transport, mitochondrial translational elongation and other BPs related to miRNAs, neuronal ribonucleoprotein granules, the mitochondrial matrix, the mitochondrial part and other Cellular Components, sequence-specific single-stranded DNA binding, single-stranded telomeric DNA binding, miRNA binding and other MFs. KEGG analysis revealed that the differentially modified peptides were enriched mainly in the spliceosome, lysine degradation and ferroptosis pathways. The subcellular localization annotation also revealed that the differentially modified peptides were expressed mainly in the cytoplasm, mitochondria and nucleus.

### 2.6. SIRT3 Alleviated DON-Induced Mitophagy

**Figure 6:**
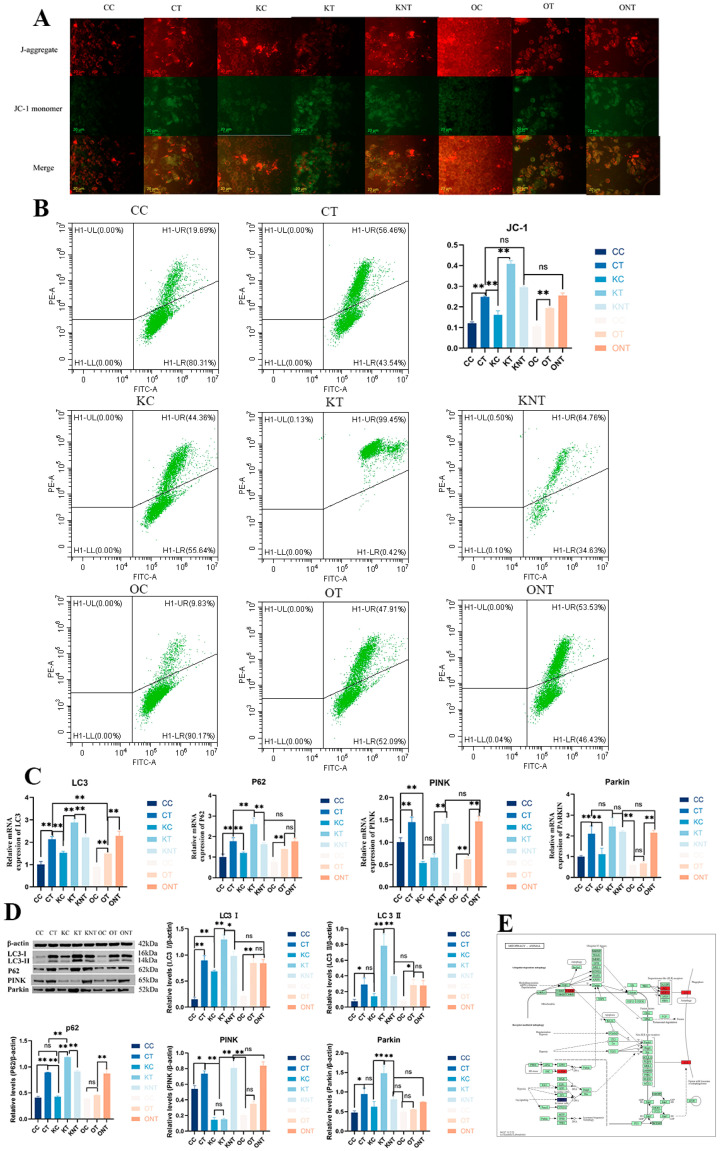
**SIRT3 alleviated DON-induced mitophagy.** (**A**). Cell membrane potential detected by fluorescence microscopy. At high mitochondrial membrane potential, JC-1 (an ideal fluorescent probe widely used to detect the ∆Ψm of mitochondrial membrane potential) could produce red fluorescence. At low mitochondrial membrane potential, JC-1 could produce green fluorescence, (**B**). Cell membrane potential detected by flow cytometry, (**C**). Gene expression of the mitophagy-related proteins LC3, P62, PINK and Parkin, (**D**). Protein expression of the mitophagy-related proteins of LC3, P62, PINK and Parkin, and a statistical analysis graph depicting these protein expression levels was generated using Image J software (1.53r) expression at the protein levels. Data are presented as mean ± SE (n = 3). Significance compared with control (ns *p* > 0.05, * *p* < 0.05 and ** *p* < 0.01). The same applies in subsequent figures. (**E**). DEPs linked to the mitophagy pathway, highlighting those upregulated in red, highlighting those downregulated in blue (n = 3).

Therefore, we next investigated the effects of DON and SIRT3 on mitophagy. We first analyzed the mitochondrial membrane potential qualitatively and quantitatively via fluorescence microscopy and flow cytometry, respectively (Figure 6A,B). The results revealed that the CT group and the KT group presented a significant decrease in mitochondrial membrane potential, and the membrane potential also decreased significantly, whereas the OT group presented significant alleviation. The proteomics results clearly revealed enrichment of the mitophagy pathway, so we validated the mitophagy pathway. The results revealed that the gene and protein levels of the autophagy marker proteins LC3 and P62 in the CT group were greater than those in the CC group, and those in the KT group were greater than those in the KC group. Compared with the OC group, the OT group had a greater percentage but was significantly lower than the CT group and KT group. Compared with the CC group, the CT group presented significant activation of the PINK1/Parkin mitophagy pathway. Compared with the KC group, the KT group presented significantly greater parkin expression, although PINK1 expression did not significantly change. Compared with the CT group and KT group, the OT group also presented significant reductions in PINK1 and Parkin expression. These results indicate that DON can significantly induce mitophagy and that the overexpression of SIRT3 can significantly alleviate this pathway.

### 2.7. SIRT3 Alleviated DON-Induced Apoptosis

**Figure 7:**
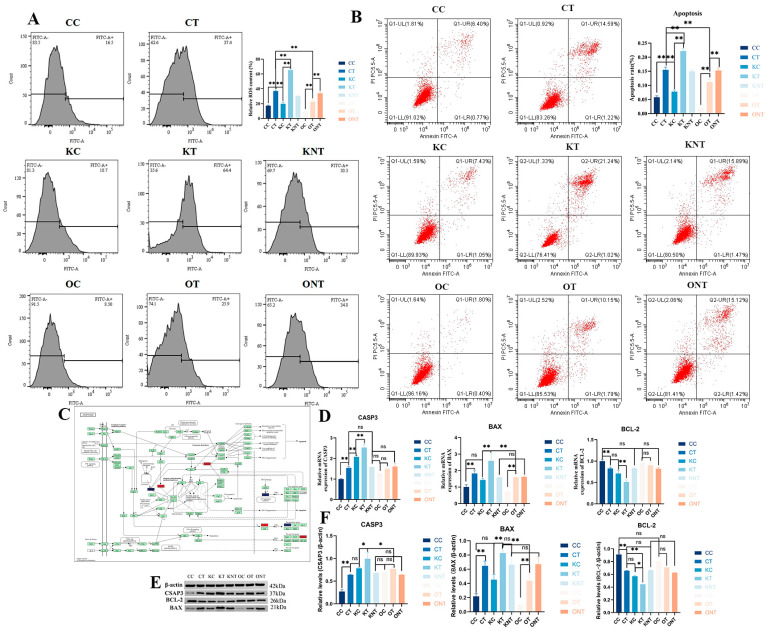
**SIRT3 alleviated DON-induced apoptosis.** (**A**). Flow cytometry for detecting cellular ROS, (**B**). Flow cytometry was used to detect apoptosis, (**C**). DEPs linked to the apoptosis pathway, highlighting those upregulated in red, highlighting those downregulated in blue, (**D**). Gene expression of the apoptosis-related proteins CASP3, BAX and BCL-2, (**E**,**F**). Protein expression of the apoptosis-related proteins CASP3, BAX and BCL-2, and a statistical analysis graph depicting these protein expression levels was generated using Image-J (1.53r) software expression at the protein levels. Data are presented as mean ± SE (n = 3). Significance compared with control (ns *p* > 0.05, * *p* < 0.05 and ** *p* < 0.01). The same applies in subsequent figures.

We also measured the content of intracellular ROS. The results showed that ROS levels were significantly higher in the CT group compared to the CC group, in the KT group compared to the KC group, and in the OT group compared to the OC group. However, the ROS level in the OT group was significantly lower than that in the CT group. Finally, the effects of DON and SIRT3 on PAM cell apoptosis were investigated. Flow cytometry was used to calculate the proportion of apoptotic cells, and the results (Figure 7) revealed that the apoptosis rates of the CT and KT groups were significantly greater than those of the CC and KC groups. Although the percentage of apoptotic cells in the OT group was greater than that in the OC group, it was significantly lower than that in the CT and KT groups. We subsequently validated the apoptotic pathway. The results revealed that at the protein and gene levels, the level of the antiapoptotic protein BCL-2 in the KT and CT groups was significantly lower than that in the KC and CC groups, the level of the proapoptotic protein BAX was significantly increased and the level of the apoptotic marker protein CASP3 was increased. The level of the antiapoptotic protein BCL-2 in the OT group was lower than that in the OC group, but not significantly, and the levels of BAX and CASP3 were also increased. Interestingly, CASP3 expression was elevated after both the knockout and overexpression of SIRT3, which may have been due to the change in acetylation status. These findings indicate that the overexpression of SIRT3 significantly alleviated DON-induced cell apoptosis, and they are consistent with the proteomic results.

## 3. Discussion

SIRT3 is a NAD^+^-dependent protein deacetylase. It plays an important role in regulating cell metabolism, antioxidative stress, aging and various diseases. In our study, SIRT3 knockout disrupted various substances, such as lipids, carboxylic acids and ketones, and various mitochondrial antioxidant functions, such as oxidoreductase activity and monooxygenase activity. After SIRT3 overexpression, the response to wounding, external stimuli, etc., is significantly improved. SIRT3 knockout leads to decreased expression of key enzymes involved in fatty acid oxidation, such as CPT1, CPT2 and LCAD [27], reduced mitochondrial energy production and aggravated cell damage. In addition, SIRT3 knockout aggravates the inflammatory response of microglia by affecting the metabolism of ketone bodies [28], and the overexpression of SIRT3 reduces neuroinflammation and oxidative damage by regulating the metabolism of ketone bodies and the activation of microglia. SIRT3 knockout mice had significantly increased levels of apoptosis in renal tubular epithelial cells [29], which promoted renal interstitial fibrosis. In SIRT3 knockout cardiomyocytes, the expression of a myocardial injury marker (cTnT) is increased [30], cell viability was decreased and cardiotoxicity is aggravated. However, the activation of SIRT3 can inhibit cardiomyocyte apoptosis and alleviate cardiac fibrosis by reducing ROS, enhancing the activities of antioxidant enzymes such as SOD2, and improving mitochondrial function [31].

However, SIRT3 is complex in various tumor types. On the one hand, SIRT3 knockout leads to disordered glutamine metabolism and induces autophagy-dependent cell death in diffuse large B-cell lymphoma (DLBCL) [32]. In glioblastoma (GBM), SIRT3 inhibition triggers mitophagy and simultaneously downregulates the expression of the ferroptosis antagonist SLC7A11 [15], sensitizing tumor cells to ferroptosis. On the other hand, a small molecule activator of SIRT3 inhibited tumor cell proliferation and migration in RKO and HCT-116 cell lines by triggering autophagy-dependent cell death and apoptosis by regulating the SIRT3/Hsp90/AKT signaling pathway [33]. In addition, overexpression of SIRT3 significantly inhibited the proliferation ability and colony formation number of gastric cancer cells by downregulating Notch-1, and inhibited the proliferation of gastric cancer cells [34]. Interestingly, both SIRT3 activation and knockout enhance viral proliferation [35,36]. SIRT3 knockout may lead to the loss of antiviral ability in the body. After SIRT3 activation, it induces the reprogramming of glutamine metabolism in host cells (such as the upregulation of glutaminase (GLS) and glutamate dehydrogenase (GDH)) and promotes the proliferation of infectious spleen and kidney necrosis virus (ISKNV) [37]. In our study, knockout and overexpression of differential SIRT3 proteins are significantly enriched in the viral protein interaction with cytokine and cytokine receptor pathways, and their application in infectious diseases needs to be evaluated with caution.

DON is a global pollutant that widely contaminates cereals and feed, mainly wheat, corn, barley, oats and other cereals and their products [38]. It has toxic effects on humans and animals, such as intestinal toxicity, immunotoxicity, neurotoxicity and reproductive and developmental toxicity [39]. In our study, DON causes 314 proteins to be upregulated and 407 proteins to be downregulated. The affected proteins are also enriched in the immune response, immune system process, regulation of cell population proliferation and other biological processes, as well as ferroptosis, mitophagy, the MAPK signaling pathway and other pathways. These results indicate that DON causes immune damage to the body through various immune pathways. After SIRT3 knockout, DON causes 519 proteins to be upregulated and 559 proteins to be downregulated. After SIRT3 overexpression, DON causes only 120 proteins to be upregulated and 119 proteins to be downregulated, but both affect metabolic processes related to fatty acids, lipids, small molecules and amino acids and other substances, as well as the activities of enzymes such as oxidoreductases and peptidases. In addition, the neuroactive ligand–receptor interaction pathway was significantly affected after SIRT3 knockout, suggesting that SIRT3 knockout aggravated the neurotoxicity caused by DON. Interestingly, the pathways affected by these differential proteins were enriched mainly in mitochondria, so we verified the effect of SIRT3 on the apoptosis pathway of the mitophagy pathway induced by DON in later experiments.

Acetylation is an important posttranslational modification (PTM) of proteins that are widely involved in life processes such as gene expression, metabolic regulation, the cell cycle and disease occurrence. Our results revealed that the knockout of SIRT3 was significantly enriched in multiple cellular processes, such as the regulation of metabolic processes, the PRR pathway and protein refolding. This indicates that SIRT3 affects the health of the body by influencing the metabolic pathways of various substances. A number of studies have shown that SIRT3 knockout can increase the acetylation level of long-chain acyl-CoA dehydrogenase (LCAD), acetyl-CoA synthetase 2 (AceCS2), isocitrate dehydrogenase 2 (IDH2) and malate dehydrogenase (MDH2) and inhibit enzyme activity, resulting in decreased fatty acid oxidation capacity and reduced TCA cycle efficiency [40]. In addition, SIRT3 directly inhibits the catalytic function of SOD2 by altering the charge distribution or conformation of its active center [41]. In addition, we found that the extrinsic apoptotic signaling pathway, late endosomal microautophagy, apoptotic mitochondrial changes and other pathways associated with cell death were significantly different after SIRT3 overexpression. These findings suggest that SIRT3 plays an important role in the regulation of mitochondrial metabolism, antioxidant defense and antiapoptotic processes.

A variety of toxins can affect biological functions by interfering with acetylation. DON can affect gene expression by interfering with histone acetylation, inhibiting protein synthesis and inducing oxidative stress. AFB1 metabolites (AFBOs) inhibit HDAC activity, resulting in an abnormal increase in histone H3K9 acetylation and the activation of proto-oncogenes (such as c-Myc) [42]. CTX activates ADP-ribosylation of the Gsα protein, enhances the activity of HATs (such as CBP) through the cAMP-PKA pathway and induces histone H4 acetylation in the promoter region of the IL-6 gene [43]. Our study revealed that DON could lead to a change in the acetylation state of mitochondrial translational elongation and proteins that target vacuoles are involved in autophagy and other processes. The metabolic processes associated with various substances after SIRT3 knockout, negative regulation of transcription by RNA polymerase I, mitochondrial translation, mitochondrial gene expression and other processes changed. The overexpression of SIRT3 is related mainly to miRNA transport, processing and other processes. Interestingly, for both DON and SIRT3 editing, the differentially expressed proteins were associated with mitochondrial gene expression, mitochondrial translation, the mitochondrial matrix and other mitochondria-related processes. These results indicate that both SIRT3 and DON can regulate the acetylation state of mitochondria. However, there are few reports about the acetylation state caused by SIRT3 in the regulation of DON. This experiment expands our understanding of how SIRT3 mediates post-translational modifications (PTMs) induced by mycotoxins in the context of immune injury. In the future, by conducting research on different acetylation sites, we will be able to further demonstrate the functional roles of these targets in the toxicity of DON.

Mitochondria, organelles involved in cell metabolism, are very sensitive to mycotoxins. DON inhibits ribosome function and reduces the synthesis of mitochondria-related proteins, leading to a decrease in the mitochondrial membrane potential (ΔΨm) and excessive generation of reactive oxygen species (ROS). ROS further damages mitochondrial DNA and the membrane structure, triggering mitophagy. DON also upregulated the expression of the proapoptotic proteins Bax and Bak-1 and inhibited the antiapoptotic protein Bcl-2, resulting in an increased Bax/Bcl-2 ratio, increased mitochondrial outer membrane permeability, release of apoptotic factors and activation of the caspase cascade, which in turn cleaves and activates the downstream effector caspase-3, leading to DNA fragmentation and apoptosis. SIRT3 can deacetylate superoxide dismutase (SOD2) and catalase (CAT), increase their ability to remove ROS in mitochondria and indirectly affect the activity of the PINK1/Parkin pathway to reduce mitochondrial damage [44]. However, the role of SIRT3 in DON-induced apoptosis via the PAM mitochondrial pathway has not been reported thus far. The results of this study revealed that the apoptosis rate of PAM cells decreased after SIRT3 overexpression. The mRNA and protein expression levels of the proapoptotic proteins Bax and CASP3 were significantly downregulated, and the mRNA and protein expression levels of the antiapoptotic protein Bcl-2 were significantly upregulated. These results indicate that SIRT3 may mitigate apoptosis, potentially through mitophagy pathway regulation. Thus, the damage caused by DON to PAM cells was alleviated. In future studies, these findings can be verified in vivo, and the role of SIRT3 in the systemic immune dysfunction caused by DON will be explored.

## 4. Materials and Methods

### 4.1. Cell Culture

Porcine alveolar 3D4/21 (PAM) macrophages were purchased from Shanghai Meiwan Biotechnology Co., Ltd. (Shanghai, China) and cultured in RPMI-1640 medium (Solarbio, Beijing, China). RPMI-1640 was supplemented with 10% fetal bovine serum (FBS, Gibco, São Paulo, Brazil), 100 IU penicillin/mL and 100 IU streptomycin/mL. DON was purchased from Yujing Technology Co., Ltd. (Shanghai, China). The purity of DON was ≥99.5%, and the concentration was 5 mg/bottle. The production batch number of DON was N6000100. In accordance with previous studies, 0.1, 0.2, 0.4, 0.8, 1.6, 3.2 and 6.4 μg/mL DON were added to the maintenance culture medium for subsequent experiments.

### 4.2. Construction of SIRT3 Gene Knockout in PAM Cells

LentiCRISPR v2 was used to construct three targeting plasmids, Z4, Z5 and Z6, and 293T cells were used for lentivirus packaging. The *Escherichia coli* strain DH5α was used to amplify the lentiviral vector and auxiliary packaging vector plasmids. When the degree of cell fusion was approximately 70%, the packaged lentivirus was subsequently used for virus infection. Thirty hours after infection, the lentivirus-infected samples were screened in a complete RPMI-1640 medium supplemented with 5 μg/mL puromycin. After screening, genomic DNA was extracted from the lentivirus-infected samples for PCR amplification of sgRNA flanking sequences and sequencing verification. Finally, the selected KO monoclonal homozygotes were expanded, and the samples were collected for PCR to detect mutations in the target region with the assistance of Chongqing Western Biomedical Technology Co., Ltd. (Chongqing, China).

### 4.3. Construction of a Stable SIRT3 Overexpression Cell Line in PAM Cells

The SIRT3 sequence was synthesized according to the SIRT3 sequence information in NCBI, and then the SIRT3 plasmid pCDH-CMV-MCS-EF1-copGFP-puro was ligated with the vector pcDH-CMV-MCS-EF1-copGFP-Puro under the action of T4 DNA ligase by restriction enzyme XbaI and NotI double digestion. The ligation products were subsequently transformed into competent cells and verified by restriction enzyme digestion. After verification, the three plasmid vectors were transfected into cells, and high titers of lentivirus concentrate were collected. Finally, the collected lentivirus was used to infect PAM cells, and the infected cells were screened with puromycin. After continuous screening and passaging for 3 passages, the PAM cells were used for subsequent experiments with the assistance of Chongqing Western Biomedical Technology Co., Ltd. (Chongqing, China).

### 4.4. CCK-8 Method

The cells were cultured at 1 × 10^4^ cells/well in a 96-well plate for 24 h and then washed once or twice with PBS. Then, 100 μL of DON solution with different concentrations was added to each well for further culture for 24 h, followed by washing with CCK-8 solution with PBS for further culture for 3 h. The OD value A was determined on a microplate reader at a wavelength of 450 nm. Four replicates were used for each treatment group.

### 4.5. Proteome Sequencing

The cells were divided into 8 groups. Detailed grouping is provided in Supplementary Table S2. An appropriate amount of SDT lysate was added to the samples for protein extraction and protein quantification via the BCA method. For each sample, 15 µg of protein was added to an appropriate amount of 5X loading buffer and subjected to SDS-PAGE and Coomassie Brilliant blue R-250 staining in a boiling water bath for 5 min. An appropriate amount of protein was taken from all samples and mixed into pooled samples, which were used as QC samples. The samples were trypsinized via the filter-aided proteome preparation (FASP) method. The peptide fragments of the enzymatically hydrolyzed samples were desalted via a C18 cartridge, and the peptide fragments were lyophilized and redissolved in 0.1% formic acid solution. The peptide concentration of the samples was determined by the OD280. After determination, DIA mass spectrometry was performed via an Astral high-resolution mass spectrometer. DIA analysis was performed using a nanoliter flow rate Vanquish Neo system for chromatographic separation, and samples separated by nanoliter HPLC were subjected to DIA mass spectrometry on an Astral high-resolution mass spectrometer (Thermo Scientific, Waltham, MA, USA). Proteome sequencing was performed by Applied Protein Technology Co., Ltd. (Shanghai, China).

### 4.6. Sequencing of the Acetylation

The experimental grouping and proteomics grouping were the same. The samples were subjected to UA lysis for protein extraction, followed by protein quantification. The sample proteins were then subjected to SDS-PAGE to separate the proteins and observe the bands. This was followed by overnight digestion using trypsin, and the peptides were desalted on a C18 column and lyophilized. Lyophilized samples were incubated with pretreated anti-AC-K antibody beads (PTMScan acetyl-Lysine Motif (Ac-K) Kit, Cell Signaling Technology, Danvers, MA, USA) to enrich for acetylated peptides. The enriched peptides were analyzed via LC-MS/MS via a timsTOF Pro mass spectrometer (Bruker, Manning Park Billerica, MA, USA) in combination with a NanoElute (Bruker Daltonics, Manning Park Billerica, MA, USA). Finally, the raw MS data of the samples were analyzed via MaxQuant software (Version 2.7.0.0) for identification and quantitative analysis. Sequencing of the acetylation group was completed by Applied Protein Technology Co., Ltd. (Shanghai, China).

### 4.7. Detection of the Mitochondrial Membrane Potential in JC-1 Cells

For one well of a six-well plate, the plates were washed once with PBS, and 1 mL of cell culture medium was added. A total of 1 mL of JC-1 working solution was added for 20 min at 37 °C. At the end of the incubation at 37 °C, the plates were washed twice with JC-1 staining buffer. A total of 2 mLs of cell culture medium containing serum and phenol red was added. The sections were observed under a fluorescence microscope.

Another cell suspension was collected, and the JC-1 staining working solution was added, mixed upside down and incubated at 37 °C for 20 min. The cells were precipitated at the end of the incubation period. After being washed twice with JC-1 staining buffer, the samples were resuspended by adding an appropriate amount of JC-1 staining buffer and then tested.

### 4.8. Cellular ROS Detection

Collect the cells and adjust the density to 1 × 10^6^/mL. Then pre-incubate them in the incubator for 30 min. Subsequently, add 10 μM DCFH-DA (a ROS fluorescent probe), incubate in the dark for 20 min, and wash twice with PBS to remove excess probe. Resuspend the cells in pre-cooled PBS and immediately run the assay for data acquisition of at least 10,000 cells. Analyze the relative positive percentage using FlowJo software™ v11 software.

### 4.9. Apoptosis Was Detected by Annexin V-FITC/PI Double Staining

The cell suspension was centrifuged and resuspended in a diluted binding buffer. The suspension was collected in a flow tube, and Annexin V-FITC was added and gently mixed. The cells were incubated at room temperature in the dark. The PI solution was added before the machine was used, the cells were resuspended in the reaction tube supplemented with PBS and the machine was used for detection.

### 4.10. RNA Extraction, Reverse Transcription and RT-qPCR Detection

Cell RNA was extracted with a SteadyPure rapid RNA extraction kit, and cDNA was synthesized with a total RNA reverse transcription kit. The primers used were designed via NCBI primers, and β-actin was used as the reference gene for qPCR. The specific information is shown in Table S2. The relative quantification was performed via the 2^−ΔΔCT^ method.

### 4.11. Western Blotting Assay

The proteins were extracted from the cells via an extraction kit, and the protein concentration was determined via a BCA protein concentration detection kit. The proteins were then separated by SDS-PAGE, followed by membrane transfer, visualization and, finally, gray value analysis. The following antibodies were used. SIRT3 polyclonal antibody 10099-1-AP, PINK1 polyclonal antibody 23274-1-AP, PARK2/Parkin polyclonal antibody 14060-1-AP, LC3 recombinant antibody 81004-1-RR, P62, SQSTM1 monoclonal antibody 66184-1-Ig, BAX polyclonal antibody 50599-2-Ig, Bcl2 polyclonal antibody 26593-1-AP and Caspase 3/p17/p19 monoclonal antibody 66470-2-Ig. All of these antibodies were from Proteintech (Rosemont, IL, USA).

### 4.12. Statistical Analysis

DIA data were processed by DIA-NN software (Version 2.2.0), and the proteins identified by database retrieval had to pass the set filtering parameter FDR < 1%. Excel was used to organize the relevant test data. SPSS 26.0 software (IBM SPSS) was used to analyze the differences in the experimental data via one-way ANOVA. The LSD method and Dunnett’s T3 method were used for multiple comparisons between groups. The test results are expressed as the means ± standard deviations (means ± SDs). The data were plotted via GraphPad Prism 9 software.

## 5. Conclusions

The proteomics results revealed that after SIRT3 knockout, various substances, such as lipids, carboxylic acids and ketone metabolism disorders, and various mitochondrial antioxidant functions, such as oxidoreductase activity and monooxygenase activity disorders, were detected. The overexpression of SIRT3 can significantly improve damage stimulation. DON can also significantly affect the metabolism of immune pathways, fatty acids and other substances, and SIRT3 knockout and overexpression can also significantly increase the metabolism of these substances. The results of the acetylation analysis revealed that SIRT3 knockout affected the positive regulation of mRNA metabolism, PRRs and other pathways, whereas the overexpression of SIRT3 affected various pathways, such as those related to cell death and thermogenesis. DON exposure significantly altered fatty acid degradation and the MAPK signaling pathway. The knockout and overexpression of SIRT3 under DON exposure enriched pathways such as the PPAR pathway and ferroptosis. The overexpression of SIRT3 attenuated DON-induced mitophagy by reducing the expression of LC3, P62 and the PINK1/Parkin mitophagy signaling pathway. In addition, SIRT3 reduced DON-induced cell apoptosis by reducing cellular ROS, as well as the expression of BAX and CASP3, and increasing the expression of BCL-2. In this study, we investigated the protein expression and acetylation of SIRT3 regulated by DON exposure in PAM cells and verified that SIRT3 could alleviate the damage caused by DON by reducing apoptosis through the mitophagy pathway. This study provides a theoretical basis for the future development of targeted drugs that promote SIRT3 expression to mitigate mitochondrial damage caused by DON.

## Data Availability

The raw research data are available and will be provided on request from the editors or reviewers.

## Supplementary Materials

The following supporting information can be downloaded at https://www.mdpi.com/article/10.3390/ijms26178222/s1.
