# Supplementary material for: SIRT3 Acetylation Regulates Mitophagy to Alleviate Deoxynivalenol-Induced Apoptosis in Porcine Alveolar Macrophages Cells
Source: Int J Mol Sci. 2025 Aug 25;26(17):8222. doi: 10.3390/ijms26178222 (PMC12428300; doi:10.3390/ijms26178222)
Supplement: Supplementary file 1 [file ijms-26-08222-s001.zip › Supplementary Material Figures.pdf]

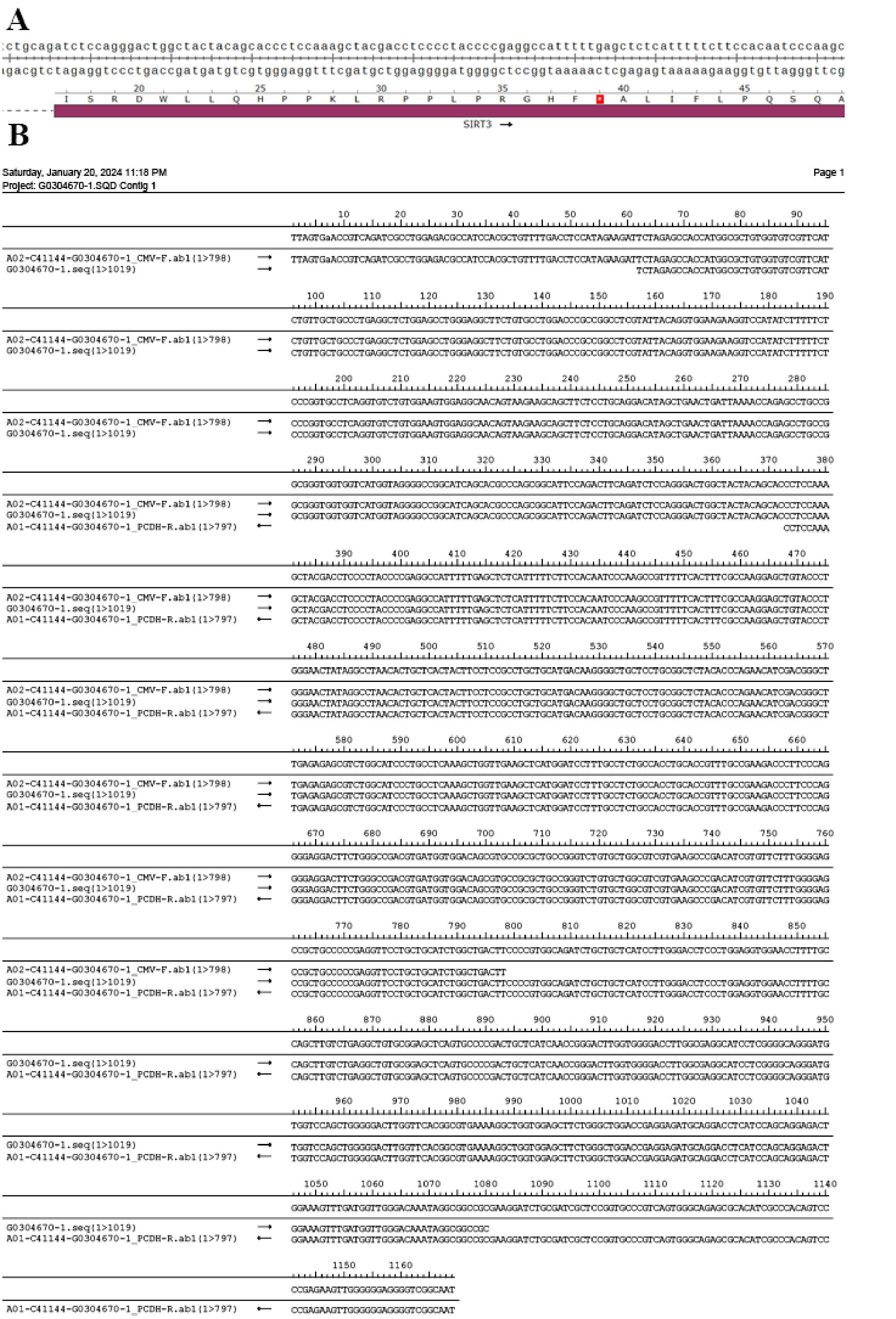

Supplement Fig. S1. Sequencing results

Note: A. Results of the first sequencing, B. Z7 pCDH-SIRT3 (puro) sequence alignment results

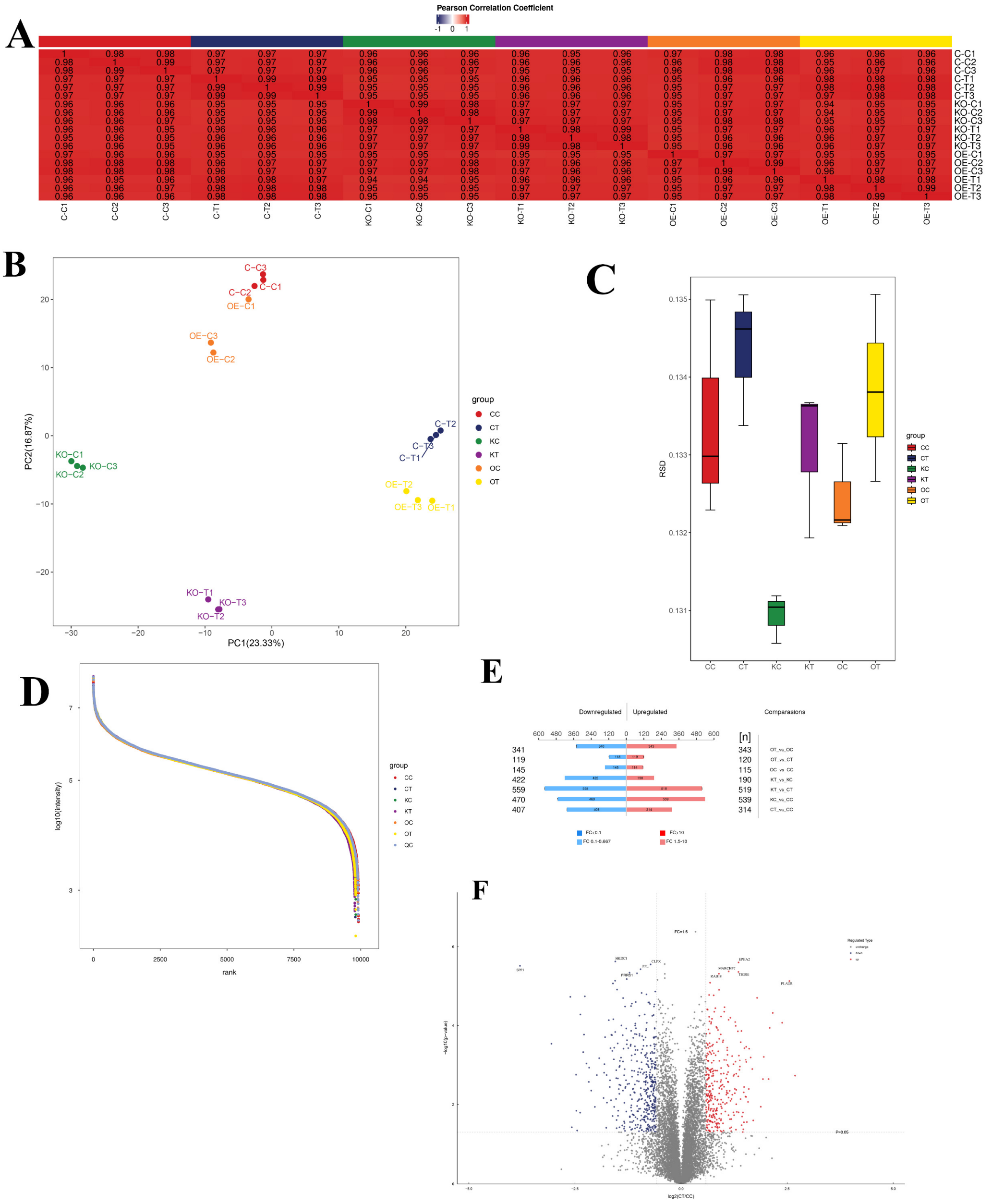

Supplement Fig.S2. The results of proteome quality control

Note: A: Map of PCC analysis of identified proteins between sample groups, When the PCC is closer to -1, it is negative correlation, closer to 1, it is positive correlation, and closer to 0, it is no correlation, B: PCA distribution map, each dot represents a sample and each color represents a different group, C: RSD analysis of identified proteins, D:Scatter plot of protein abundance distribution, E: Bar graph of protein quantitative difference results, F:Volcano map of CT vs CC group, In the figure, the red points are

the up-regulated significantly differentially expressed proteins, the blue points are the down-regulated significantly differentially expressed proteins, and the gray points are the proteins with no difference change. The points marked with ID are the most significantly different top5 up-and down-regulated proteins.

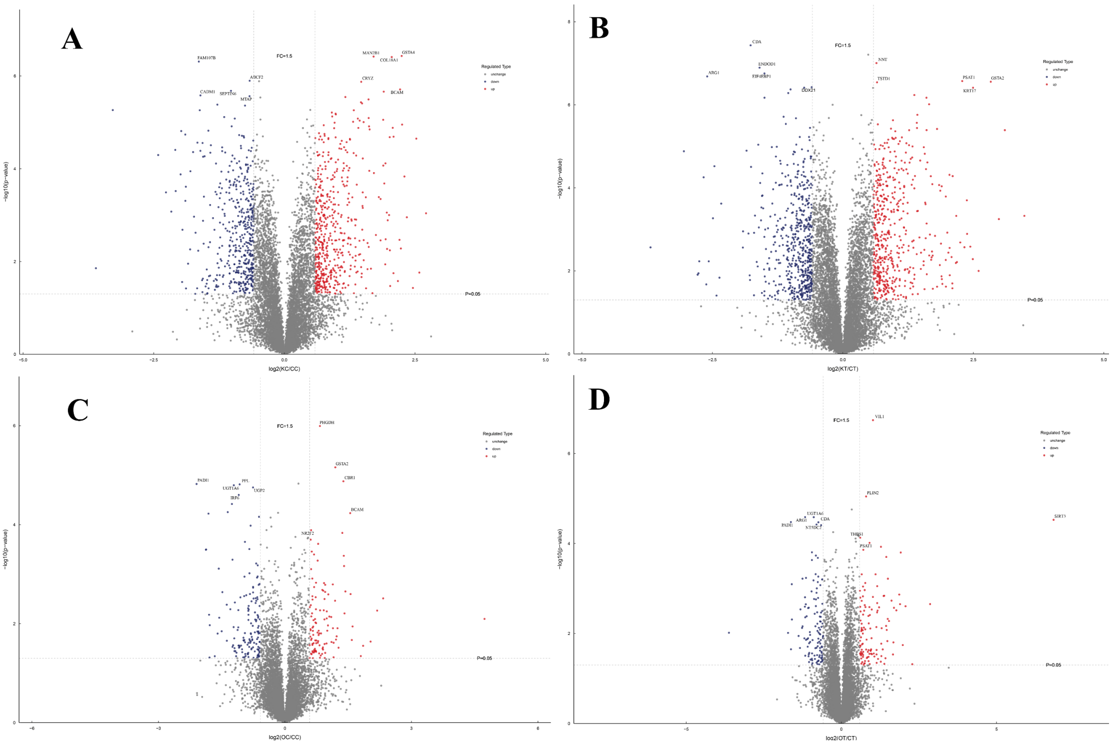

Supplement Fig.S3. Volcano plot of DEPs

Note: A: OC vs CC, B: CT vs CC, C: KT vs CT, D: OT vs CT

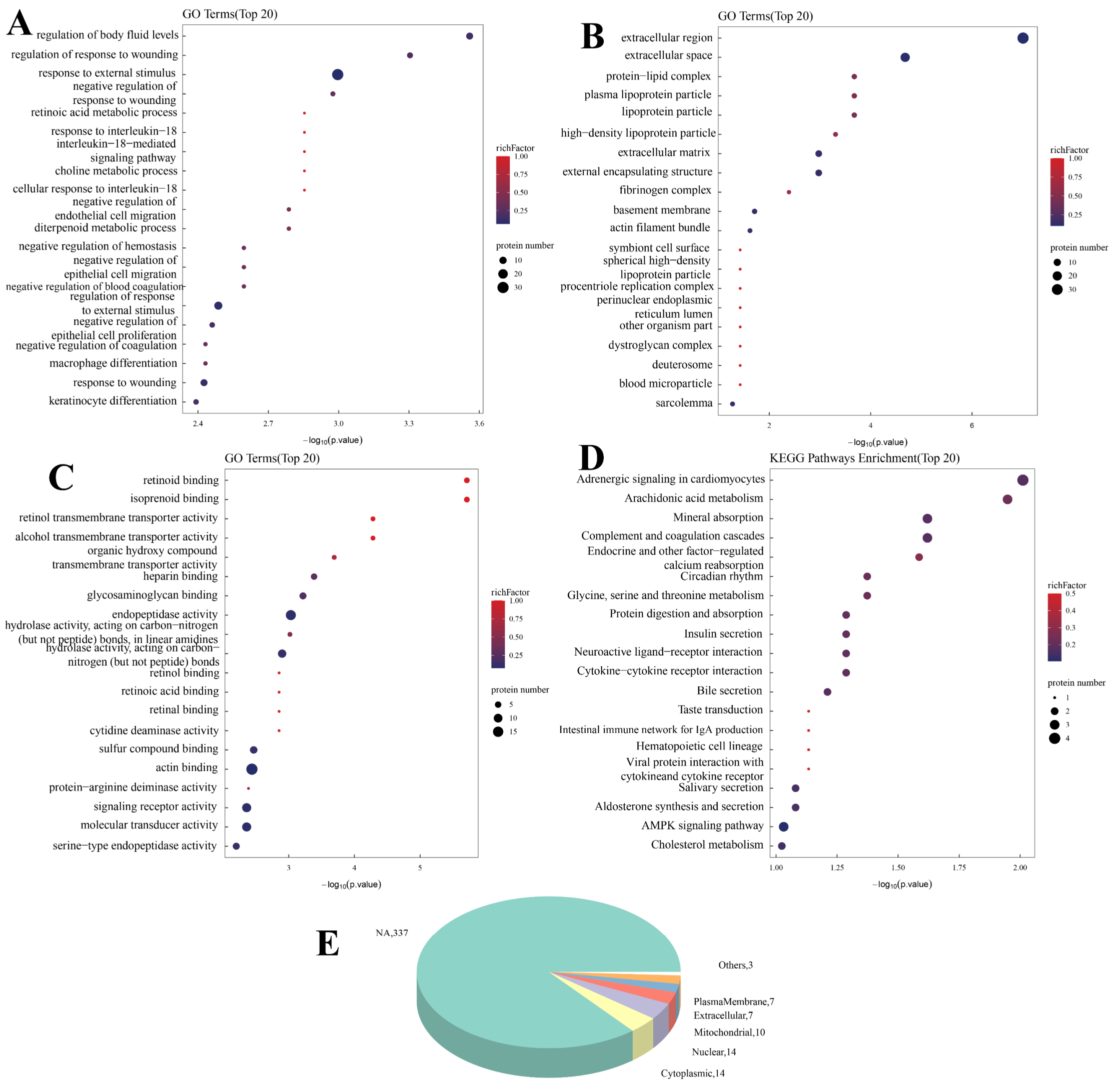

Supplement Fig.S4. Bioinformatics analysis of intracellular differential proteins in PAM under the influence of overexpression SIRT3 (OC vs CC)

Note: A: GO-BP enrichment bubble plot (OC vs CC), B: GO-CC enrichment bubble plot (OC vs CC), C: GO-MF enrichment bubble plot (OC vs CC). D: KEGG pathway classification map (OC vs CC). E: Pie chart of subcellular localization distribution. Enriched GO terms with a *p*-value of < 0.05 were exhibited.

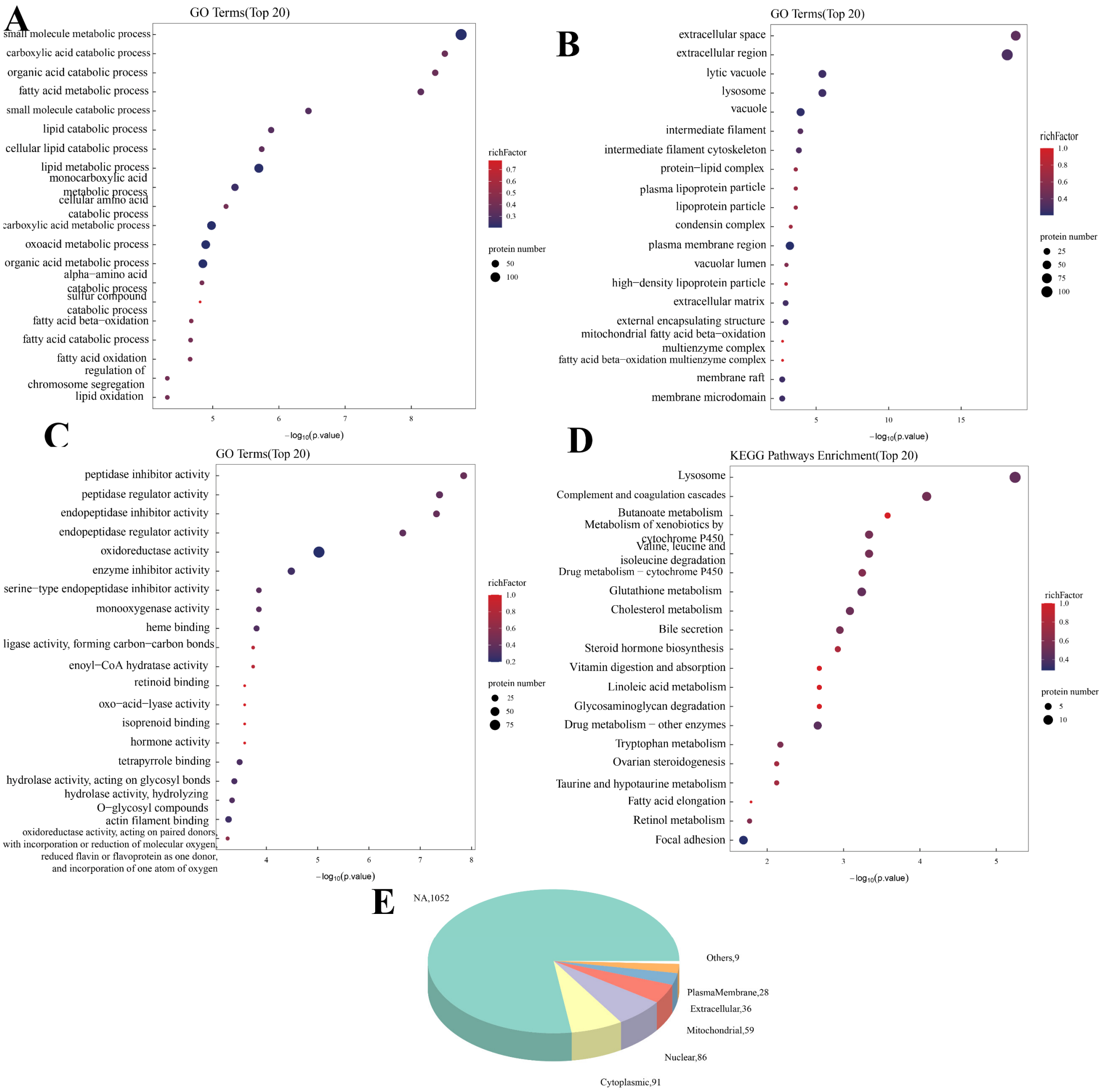

Supplement Fig.S5. Bioinformatics analysis of intracellular differential proteins in PAM under the influence of knockout SIRT3 and DON (KT vs CT)

Note: A: GO-BP enrichment bubble plot (KT vs CT), B: GO-CC enrichment bubble plot (KT vs CT), C: GO-MF enrichment bubble plot (KT vs CT). D: KEGG pathway classification map (KT vs CT). E: Pie chart of subcellular localization distribution. Enriched GO terms with a *p*-value of < 0.05 were exhibited.

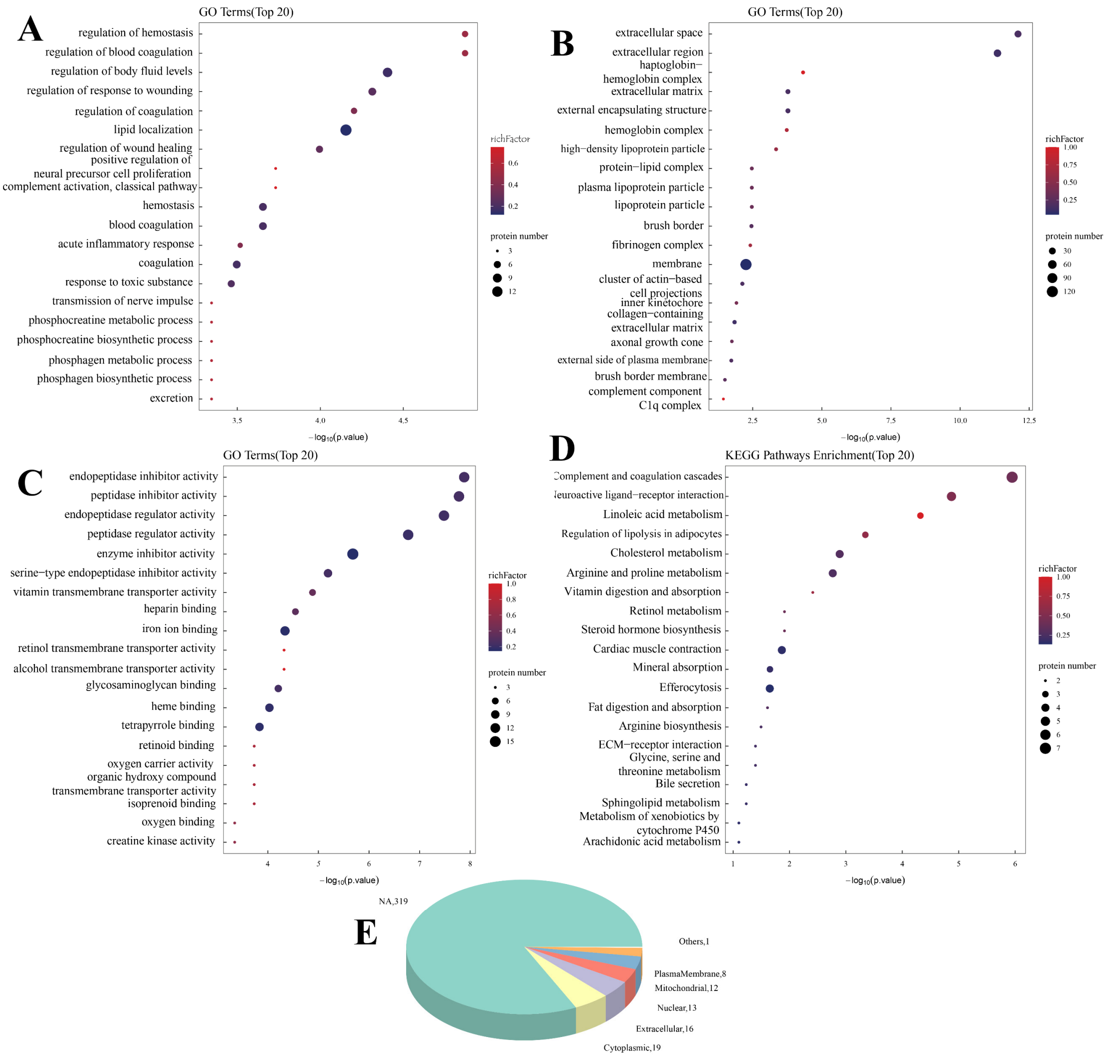

Supplement Fig.S6. Bioinformatics analysis of intracellular differential proteins in PAM under the influence of overexpression SIRT3 and DON (OT vs CT)

Note: A: GO-BP enrichment bubble plot (OT vs CT), B: GO-CC enrichment bubble plot (OT vs CT), C: GO-MF enrichment bubble plot (OT vs CT). D: KEGG pathway classification map (OT vs CT). E: Pie chart of subcellular localization distribution. Enriched GO terms with a *p*-value of < 0.05 were exhibited.

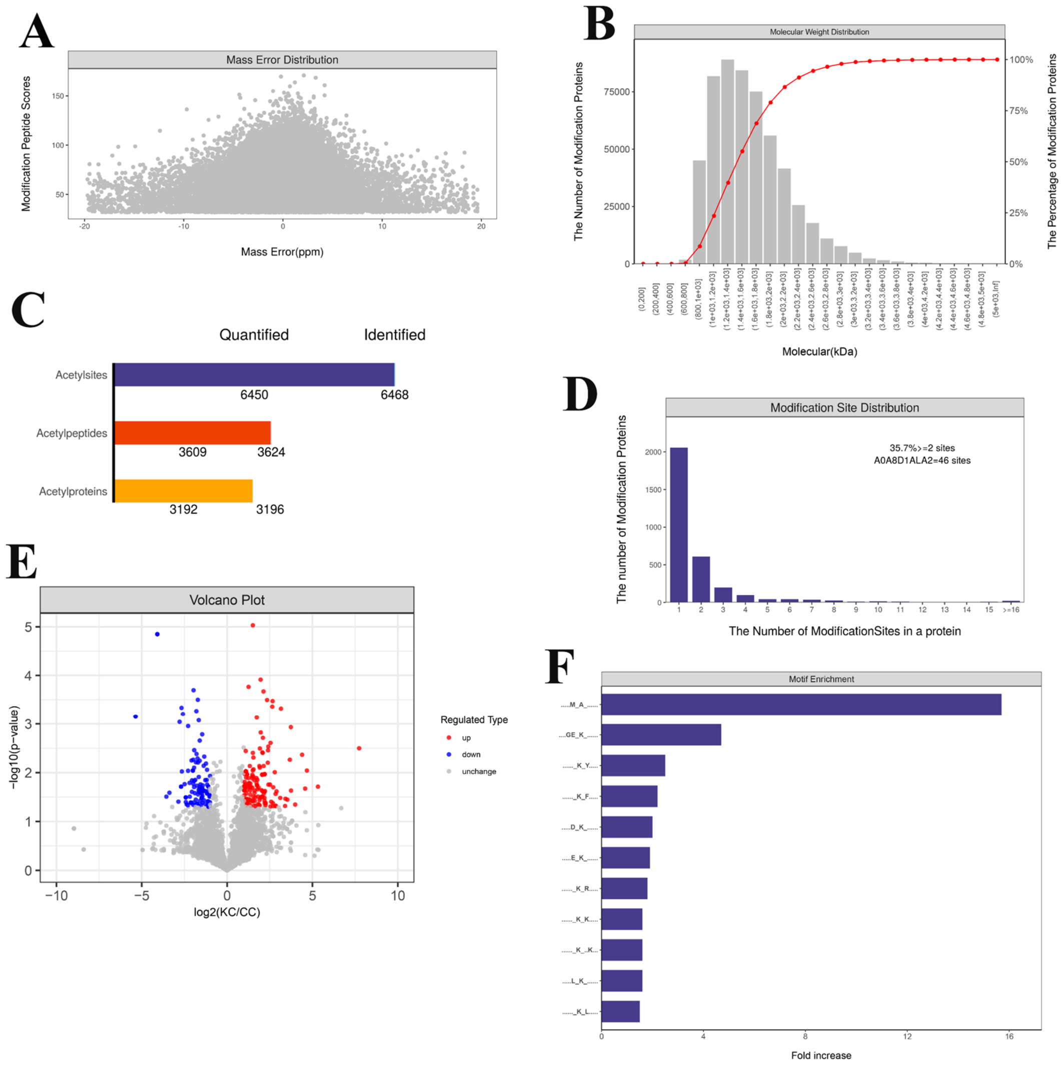

Supplement Fig.S7. Quality control results of the acetylated modified group

Note: A: Distribution of ion mass deviations of modified peptides, B: Distribution of ion scores of modified peptides. C: Statistical plots of identification and quantification results. D: Plot of the number distribution of acetylation modification sites. E: Volcano map of KC vs CC group. In the figure, the red points are the up-regulated significantly differentially expressed modified peptides, the blue points are the down-regulated significantly differentially expressed modified peptides, and the gray points are the modified peptides with no difference change. F: Statistical plots of predicted conserved motif enrichment

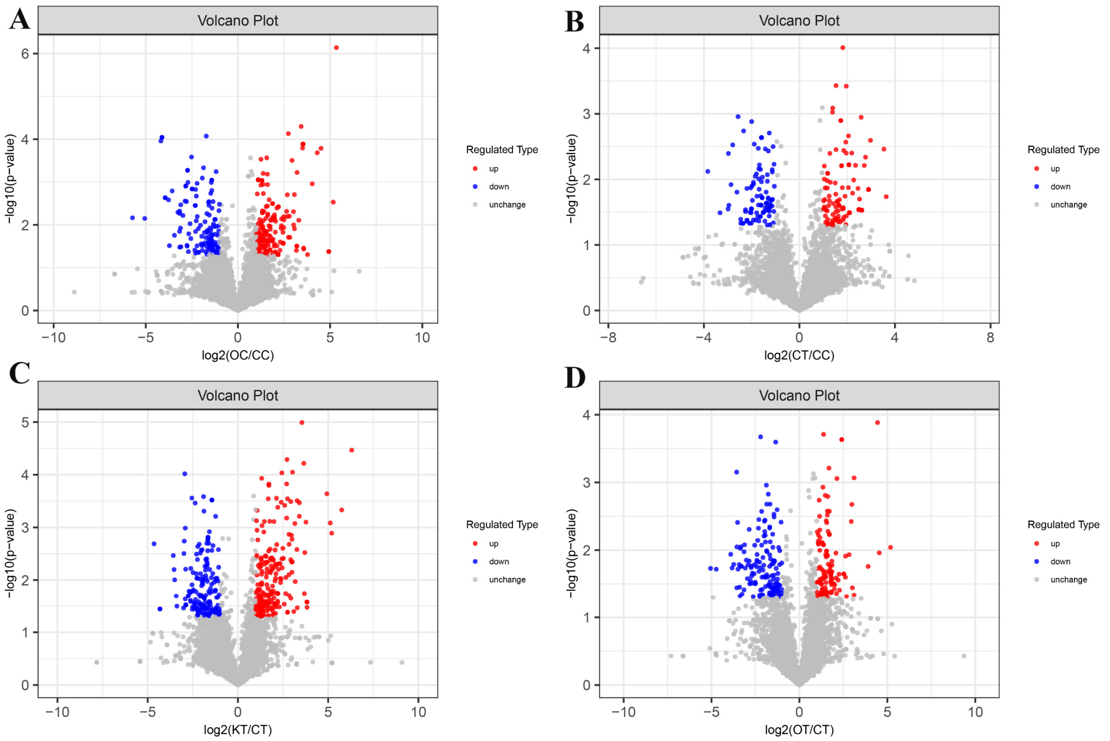

Supplement Fig.S8. Volcano plot of differentially modified peptides

Note: A: OC vs CC, B: CT vs CC, C: KT vs CT, D: OT vs CT

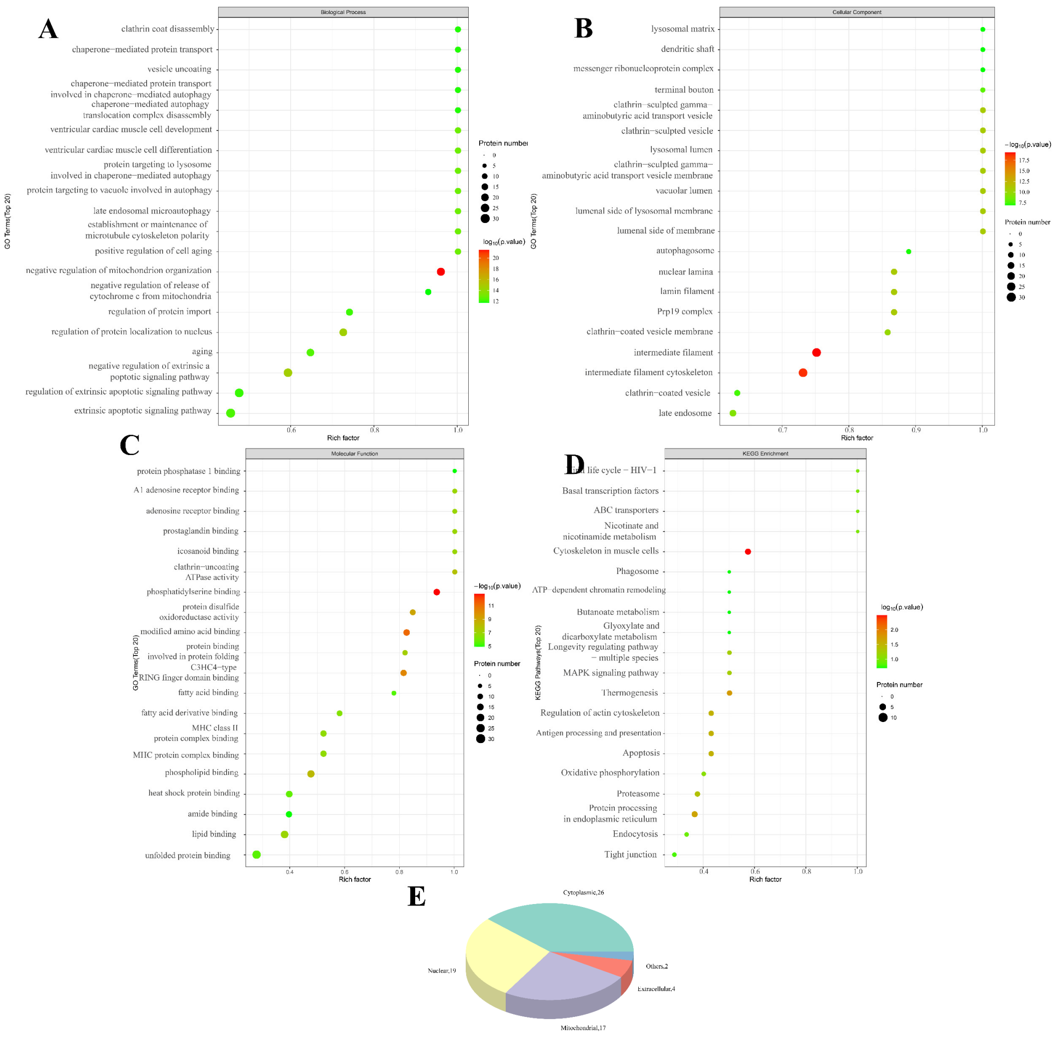

Supplement Fig.S9. Bioinformatics analysis of intracellular differentially modified acetylated peptides of PAM under the influence of overexpression SIRT3 (OC vs CC)

Note: A: GO-BP enrichment bubble plot (OC vs CC), B: GO-CC enrichment bubble plot (OC vs CC), C: GO-MF enrichment bubble plot (OC vs CC). D: KEGG pathway classification map (OC vs CC). E: Pie chart of subcellular localization distribution. Enriched GO terms with a *p*-value of < 0.05 were exhibited.

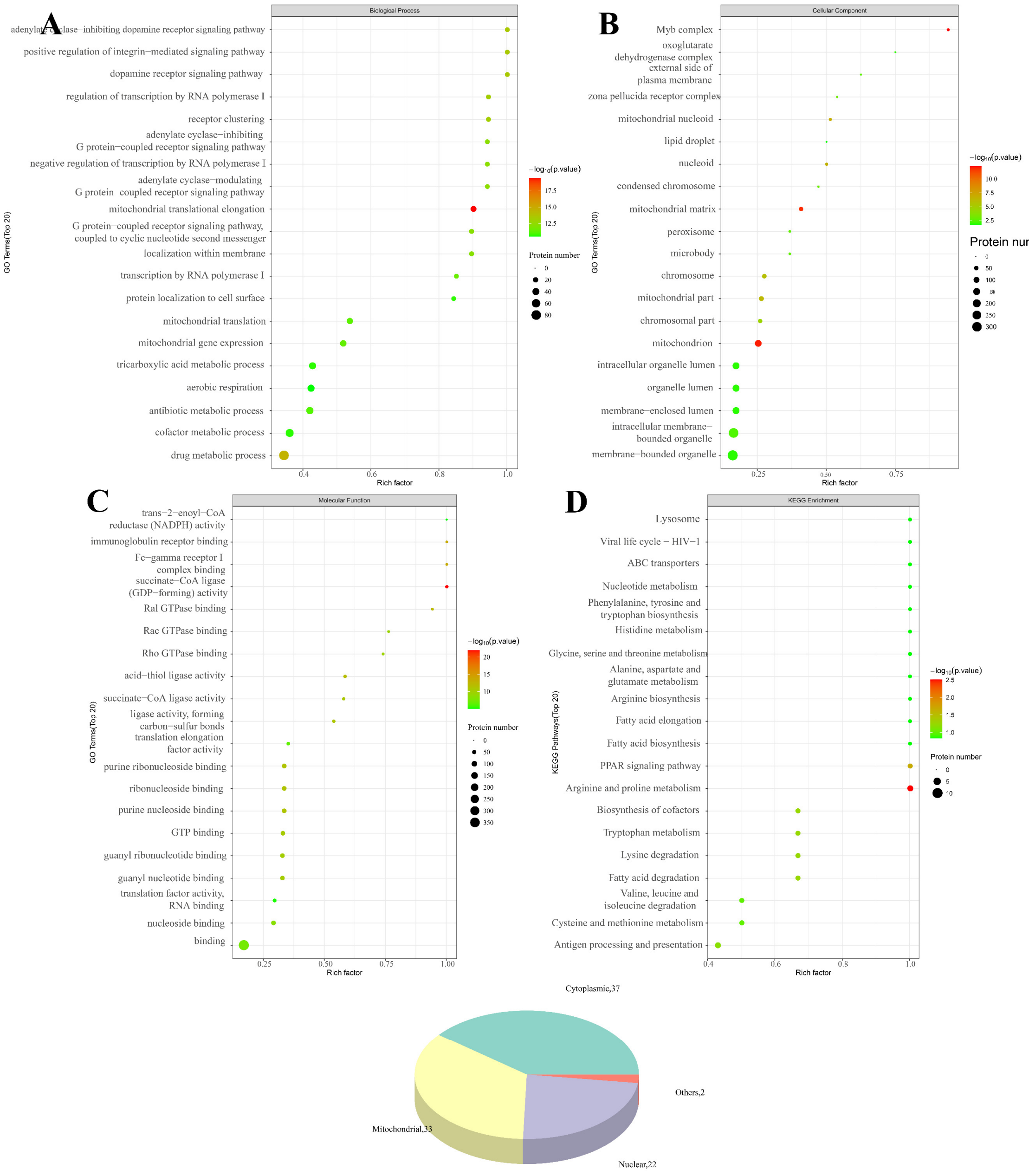

Supplement Fig.S10. Bioinformatics analysis of intracellular differentially modified acetylated peptides of PAM under the influence of knockout SIRT3 and DON (KT vs CT)

Note: A: GO-BP enrichment bubble plot (KT vs CT), B: GO-CC enrichment bubble plot (KT vs CT), C: GO-MF enrichment bubble plot (KT vs CT). D: KEGG pathway classification map (KT vs CT). E: Pie chart of subcellular localization distribution. Enriched GO terms with a *p*-value of < 0.05 were exhibited.

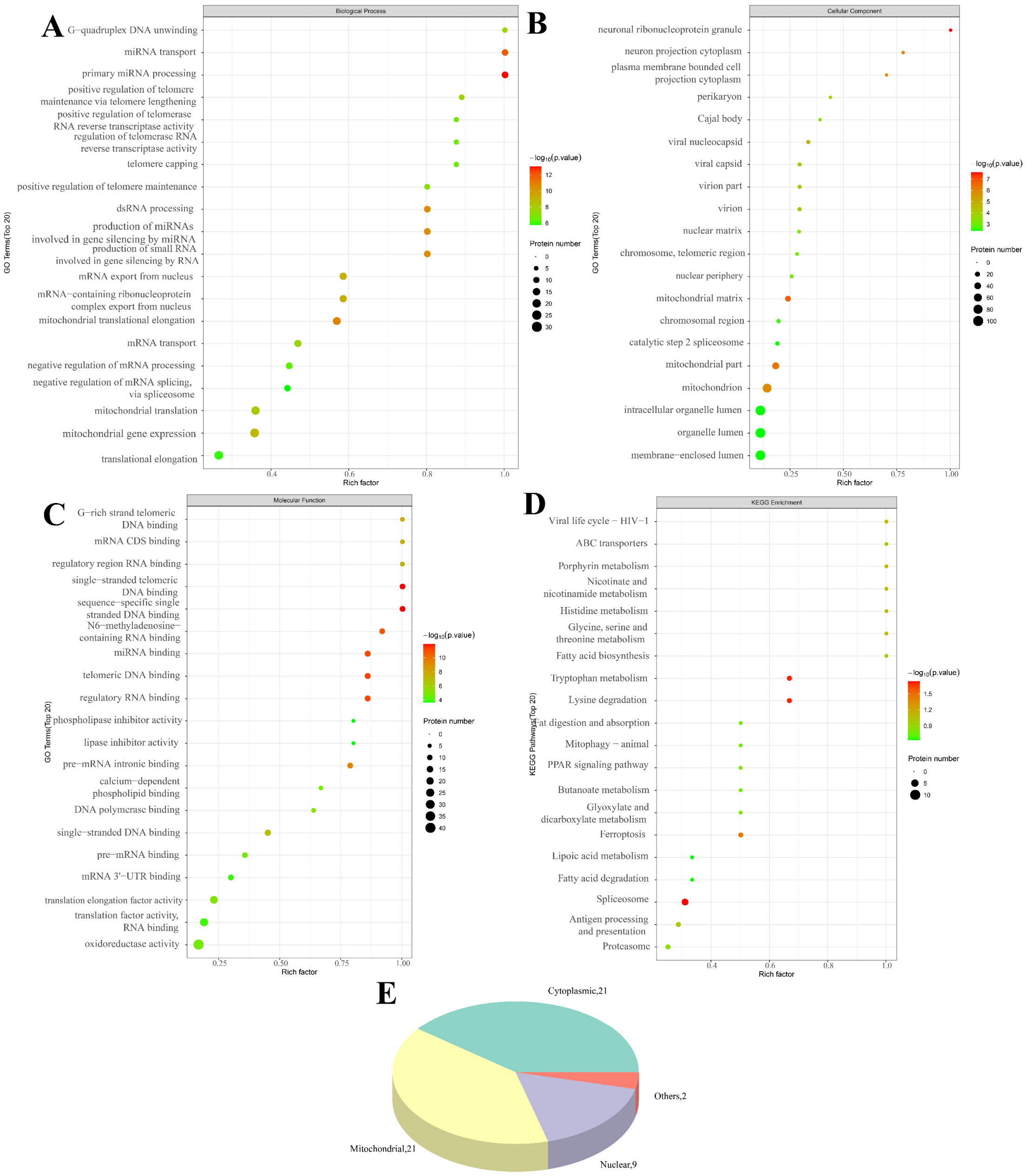

Supplement Fig.S11. Bioinformatics analysis of intracellular differentially modified acetylated peptides of PAM under the influence of overexpression SIRT3 and DON (OT vs CT)

Note: A: GO-BP enrichment bubble plot (OT vs CT), B: GO-CC enrichment bubble plot (OT vs CT), C: GO-MF enrichment bubble plot (OT vs CT). D: KEGG pathway classification map (OT vs CT). E: Pie chart of subcellular localization distribution. Enriched GO terms with a *p*-value of < 0.05 were exhibited.
