# Supplementary material for: SIRT3 Acetylation Regulates Mitophagy to Alleviate Deoxynivalenol-Induced Apoptosis in Porcine Alveolar Macrophages Cells
Source: Int J Mol Sci. 2025 Aug 25;26(17):8222. doi: 10.3390/ijms26178222 (PMC12428300; doi:10.3390/ijms26178222)
Supplement: Supplementary file 1 [file ijms-26-08222-s001.zip › Supplementary Tables.pdf]

Supplement Table S1. Grouping and Processing.

Groups	Treatment
CC group	control cells + control treatment
CT group	control cells + toxin treatment
KC group	knockout cells + control treatment
KT group	knockout cells+ toxin treatment
KNT group	knockout NC cells + toxin treatment
OC group	overexpressing cells + control treatment
OT group	overexpressing+ toxin treatment
ONT group	overexpressing NC cells + toxin treatment

Supplement Table S2. Primer sequence.

Gene ID	Login Number	Sequence (5'-3')	ProdSize
β-actin	XM_003124280.5	F: CTGCGGCATCCACGAAACT<br>R: AGGGCCGTGATCTCCTTCTG	147
SIRT3	XM_005652976.3	F: CCAGCGGCATTCCAGACTTCAG<br>R: CCCCAGGCTCAGTTGCTAA	150
LC3	NM_001170827.1	F: GCCTTCTTCCTGCTGGTGAACC<br>R: GGGAGGCGTAGACCATGTAGAGG	115
P62	XM_003123639.4	F: CTGATGAAGGTGGCTGGCTGAC<br>R: CAAGGGCGGTGGGTGTTTCG	101
PINK1	XM_021095478.1	F: TAGACGCTGGCAGGGCTTCC<br>R: TCTTCGCCGCCTCCAGGTTC	125

Parkin	NM_001044603.2	F: GTGGTTCTGCGGTGGATTCTGAG<br>R: ACTGCCTGTGGTTCTTTGGGAAG	102
BAX	XM_003127290.5	F: ATCGGCTGCTGGGCTGGATC<br>R: ATGGTGAGCGAGGCGGTGAG	124
BCL-2	XM_021099593.1	F: TCAGGGAACAGGACGCTCAGAC<br>R: CTTGGCAGGATAGCAGCACAGG	127
CASP3	NM_214131.1	F: GTGGGATTGAGACGGACAGTGG<br>R: TTCGCCAGGAATAGTAACCAGGTG	114
